# Associations of visceral adipose tissue with bone mineral density and fracture: observational and Mendelian randomization studies

**DOI:** 10.1186/s12986-022-00680-6

**Published:** 2022-07-12

**Authors:** Jianying Hu, Manying Zhao, Chenhao Lin, Zhonghan Sun, Guo-Chong Chen, Zhendong Mei, Yan Zheng

**Affiliations:** 1 State Key Laboratory of Genetic Engineering, Human Phenome Institute, School of Life Sciences, Fudan University, 2005 Songhu Road, Shanghai, 200438 China; 2 Ministry of Education Key Laboratory of Contemporary Anthropology, Human Phenome Institute, Fudan University, Shanghai, China; 3 Department of Nutrition and Food Hygiene, School of Public Health, Soochow University, Suzhou, China; 4 Ministry of Education Key Laboratory of Public Health Safety, School of Public Health, Fudan University, Shanghai, China; 5 National Clinical Research Center for Aging and Medicine, Huashan Hospital, Fudan University, Shanghai, China

**Keywords:** Visceral adipose tissue, Bone mineral density, Fracture, Mendelian randomization, UK Biobank

## Abstract

**Background:**

The associations between visceral adipose tissue (VAT) and bone mineral density (BMD) or fracture have been controversial and the causality of the associations remains to be assessed. This study aimed to explore the associations of VAT^ (predicted value of VAT mass) with BMD and fracture risk in men and women, and to examine their potential causation by two-sample Mendelian randomization (MR) analyses.

**Methods:**

UK Biobank is a large, population-based prospective cohort study that recruited more than 500,000 participants aged 40–69 in the United Kingdom from 2006 to 2010. In this study, we used a validated and reliable prediction model to estimate the VAT amount of the participants. On this basis, linear and nonlinear multivariable statistical models were used to explore the association of VAT^ with BMD and fracture risk in different groups of sex and BMI. In observational analyses, the multivariable linear regression model and Cox proportional-hazards model were used to assess VAT^ association with BMD and fracture risk, respectively. Inverse variance weighting was used as the main result of MR analysis.

**Results:**

In 190,836 men, an inverted U-shaped association was observed between VAT^ and heel BMD (*P* for nonlinearity < 0.001), with a turning point of VAT^ = 1.25 kg. Per kg increase in VAT^ was associated with a 0.13 standard deviation (SD) increase in heel BMD (*P* = 1.5 × 10^−16^) among men with lower amounts of VAT^, and associated with a 0.05 SD decrease in heel BMD (*P* = 1.3 × 10^−15^) among men with higher amounts of VAT^. In 193,592 women, per kg increase in VAT^ was monotonically associated with a 0.16 SD increase in heel BMD (*P* = 1.2 × 10^−136^, *P* for VAT^-sex interaction = 8.4 × 10^−51^). During a median follow-up of 8.2 years, VAT^ was associated with lower risks of hip fractures in the overall men and women (*P* for VAT^-sex interaction = 1.9 × 10^−4^ for total fractures; 1.5 × 10^−4^ for other fractures). There were significant interactions of VAT^ and BMI on heel BMD and fracture risks in men only (*P* for VAT^-BMI interaction = 5.9 × 10^−31^ for heel BMD; 2.7 × 10^−4^ for total fractures; 5.7 × 10^−3^ for hip fractures; 6.8 × 10^−3^ for other fractures). In two-sample MR analyses, evidence of causality was not observed between VAT^ and DXA-derived BMD or fractures.

**Conclusions:**

These novel findings demonstrated gender-dependent associations of VAT^ with BMD and fracture risk, with the association in men being modified by adiposity. Evidence of causality was not observed, suggesting that the observational association of VAT^ with BMD and fracture risk could be the result of confounding.

**Supplementary Information:**

The online version contains supplementary material available at 10.1186/s12986-022-00680-6.

## Introduction

Osteoporosis is the most common metabolic bone disease characterized by low bone mineral density (BMD), which is a strong risk factor for fracture among older adults [1]. Approximately 50% of women and 20% of men will suffer from an osteoporotic fracture in their lifetime [2]. Obesity, usually defined by body mass index (BMI), has been reported to be associated with higher BMD and lower risk of fractures [3–5]. However, BMI as a measure of obesity has been criticized for its limited capacity to differentiate body fat mass and body lean mass, [6] and the latter has been consistently shown to be favorably associated with higher BMD [7, 8]. By contrast, the associations of body fat mass, especially the visceral adipose tissue (VAT), with BMD and fracture risk are controversial [9–12].

VAT refers to body fat stored in the abdominal cavity [13]. Results from a few studies on VAT and skeletal health are inconsistent [9–12]. A cross-sectional study suggested that abdominal fat mass was positively associated with femoral neck BMD, but not with lumbar spine BMD [9]. A recent study reported that higher amounts of VAT were associated with greater BMD and better microstructure of the peripheral skeleton [14]. Most of these reported associations were only observed in women, and were no longer significant when adjusting for BMI or weight [14]. In addition, a case-cohort study suggested no significant relationship between VAT and incident fractures [12]. Furthermore, the causal relationships between VAT and skeletal outcomes remain unclear. Mendelian randomization (MR) uses genetic variants as instrumental variables for an exposure (e.g. VAT) to infer causality [15]. The MR design was less susceptible to bias due to confounding factors or reverse causation than observational studies [16].

In the present study, we adopted a VAT prediction model [17] to estimate the associations of the predicted VAT mass (VAT^) with BMD and fracture risk by gender and adiposity status among 395,612 White British participants from the UK Biobank cohort. Furthermore, we examined the potential causation for such associations by using two-sample MR analyses.

## Methods

### Observational analysis

#### Study population

UK Biobank is a prospective cohort study that recruited more than half a million participants aged 40–69 years in the United Kingdom between 2006 and 2010 [18]. At baseline, participants were assessed for their demographic characteristics, lifestyle, medical histories, and women’s reproductive factors, through touchscreen questionnaires and oral interviews. In addition, they underwent various physical examinations such as measurements of weight, height, waist circumference, and hip circumference. Bioelectrical impedance was measured by the Tanita BC418MA body composition analyzer, which uses an eight-electrode setup that estimates body composition in the whole body as well as in the limbs [17]. VAT mass was measured by dual-energy X-ray absorptiometry (DXA) in a subset of 5,109 individuals using the GE Healthcare Lunar iDXA scanner.

#### Predicted values of VAT mass

Using the methods developed by Karlsson T et al., we estimated the predicted measurements of VAT mass (VAT^) from the multivariable-adjusted model, for men and women separately [17]. The following variables were included in the prediction model: age, menopause status, height, weight, waist circumference, hip circumference, impedance of left arm, impedance of right arm, impedance of left leg, impedance of right leg, and impedance of the whole body. The training dataset included 5,109 participants with VAT mass measured by DXA, and the application dataset included the whole study sample of the baseline. To avoid potential bias due to population structure, only White British participants were included in the study, and those who had ‘Do not know’, ‘Prefer not to answer’ or ‘NA’ variables in the prediction model were set as missing the corresponding variables [21]. After removal, 396,403 participants passed the initial quality control and obtained the VAT^, and the following participants were further excluded from the analysis: (1) individuals with extreme waist (≤ 26 cm) or hip circumference compared to their body size measurements (hip > 2 × BMI + 80 cm); (2) individuals with a difference in impedance between the left and right arm or the left and right leg of more than 120Ω. Detailed information about the exclusion criterion of participants has been published elsewhere [17]. In total, 395,612 participants constituted the final application dataset (Additional File 2: Fig. S1).

#### Skeletal outcomes

**Fig. 1:**
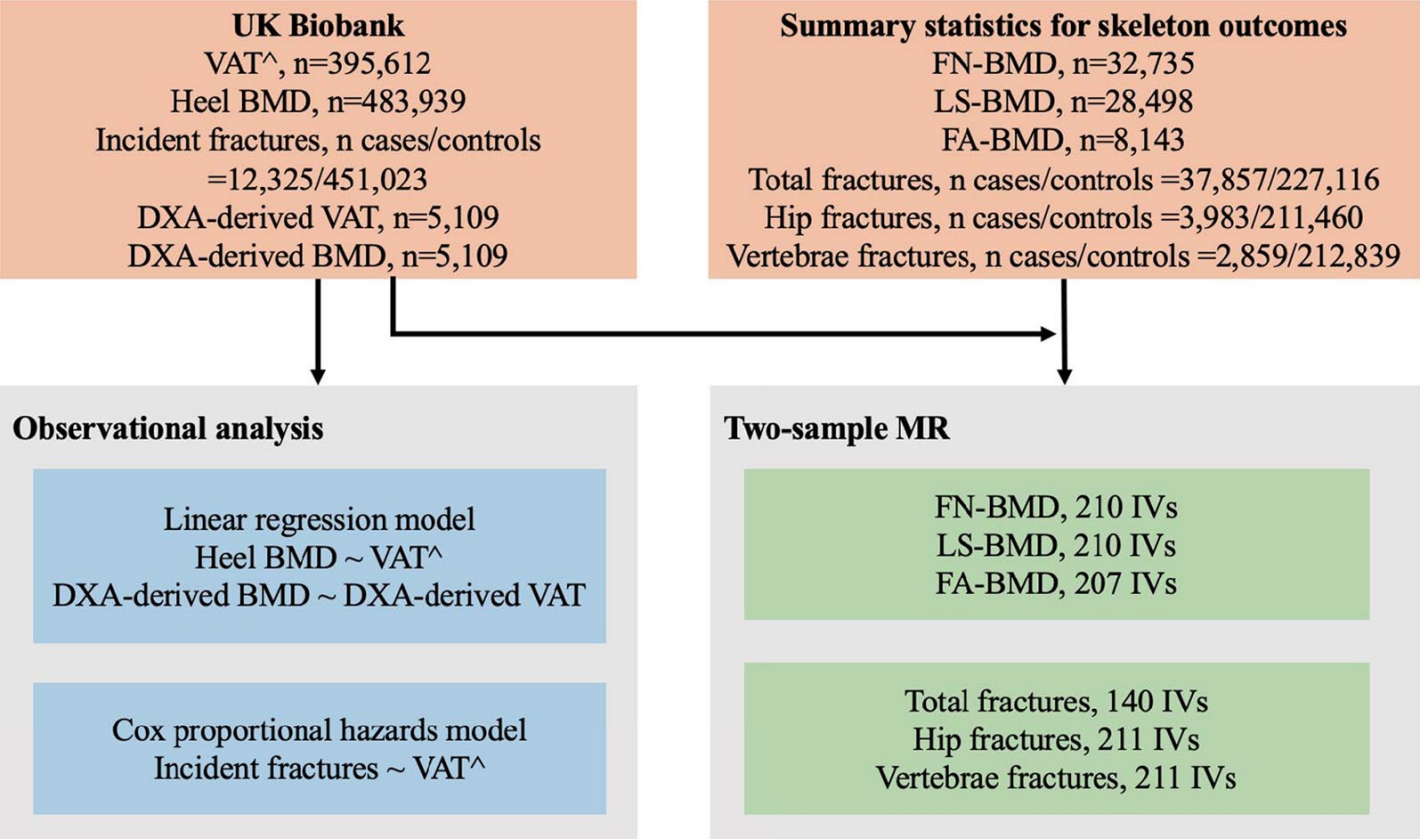
Flow diagram of study design. *VAT*^, predicted values of VAT (visceral adipose tissue) mass; *BMD* bone mineral density; *DXA* dual-energy x-ray absorptiometry; *FN-BMD* femoral neck BMD; *LS-BMD* lumbar spine BMD; *FA-BMD* forearm BMD; *MR* mendelian randomization; and *IV* instrumental variable

Heel BMD was estimated based on ultrasound measurement of the calcaneus during baseline assessment, using a Sahara Clinical Bone Sonometer (Hologic Corporation, Bedford, MA, USA) [19]. After quality control, a total of 483,939 individuals were included in the analysis (Fig. 1). Incident fractures were identified according to the ICD-10 codes at hospital admissions (Additional file 1: Table S3) [19]. Participants with fractures of the skull, face, hands, and feet (usually due to trauma), pathological fractures (due to malignant tumors), atypical femoral fractures, periprosthetic fractures, and fracture healing were excluded from the analysis [19]. Traumatic fractures were not excluded because the cause of the trauma was not well captured by the ICD-10 codes. Fractures at the hip and vertebrae are most likely attributed to low BMD [20, 21]. Therefore, hip, vertebrae, and other fractures were included as outcomes for specific sites of fractures in this study. The date of fracture was determined from hospital admission data. Date of death was determined from death certificates held by the National Health Service (NHS) Information Centre for participants from England and Wales, and the NHS Central Register Scotland for participants from Scotland [18]. In the current analysis, hospital admission data were available until March 31, 2017, and mortality data were available until February 14, 2018. Therefore, we censored follow-up on March 31, 2017, or the date of first fracture or death, whichever occurred first. In total, 451,023 participants without baseline fractures were included in the analysis (Fig. 1).

#### Covariates

Baseline touchscreen questionnaires were used to assess several potential confounding variables, including age, household income, smoking status, alcohol consumption, physical activity, dietary supplements, overall health rating, medical history including the histories of type 2 diabetes (T2D), cardiovascular disease (CVD), or cancer diagnosed by a doctor, and menopausal status and hormone replacement therapy (women only). Lean mass and body weight were measured with the Tanita BC-418 MA body composition analyzer (Tanita Corporation of America, IL). Standing height was measured using the Seca 202 device (SECA, Hamburg, Germany). BMI was calculated as weight in kilograms divided by height in meters squared.

#### Statistical analysis

According to gender-specific quartiles of VAT^, baseline characteristics were presented as percentage (%) for categorical variables and mean (standard deviation, SD) for continuous variables, for men and women separately. Heel BMD was normalized using the R ‘scale’ function [22] before analysis. VAT^ was examined as a continuous variable with the unit kg in all models. The regression coefficient (β) and standard error (SE) for the association of BMD with VAT^ were assessed by using the linear regression models. The hazard ratios (HRs) and 95% confidence interval (CI) for fracture risk associated with VAT^ were assessed by using the Cox proportional hazards models. The proportional hazards assumption of Cox models was tested based on the Schoenfeld residuals and almost no evidence of violation was observed (Additional file 1: Table S9). We also used restricted cubic splines with five knots at the 5th, 25th, 50th, 75th, and 95th centiles to flexibly model the association of VAT^ with heel BMD and fracture risk. We tested for potential nonlinearity by using a likelihood ratio test comparing the model with only a linear term against the model with linear and cubic spline terms [23, 24]. All models were adjusted for age (years), annual household income (< £18,000, £18,000-£30,999, £31,000-£51,999, £52,000-£100,000, or > £100,000), lean mass (kg), standing height (cm), smoking status (never, previous, or current), alcohol consumption (less than 3 times/month, 1–2 times/week, or more than 3 times/week), physical activity (metabolic equivalent task [MET] -min/week), intakes of calcium and vitamin D supplements (both as yes or no), overall health rating (excellent, good, fair, or poor), baseline disease history including T2D, CVD, and cancer (all as yes or no), and for women, menopause status (premenopausal or postmenopausal) and use of hormone replacement therapy (yes or no). All analyses were conducted by gender (men and women), and we further grouped men and women by BMI (< 25 kg/m^2^ and ≥ 25 kg/m^2^) in these observational analyses. The interactions of sex and BMI with VAT^ on skeletal outcomes were estimated by including a multiplicative factor in all models. To minimize the potential confounding effects and reverse causality brought by diabetes, CVD, and cancer [25–29], we also conducted additional sensitivity analyses by excluding participants who had self-reported physician-diagnosed T2D, CVD, or cancer at baseline. *P* < 0.05 (two-tailed) was considered to be statistically significant. All analyses were performed using R (version 3.6.1).

### Mendelian randomization analysis

In the main analyses, we performed two-sample MR analyses (Fig. 1) using individual-level data from UK Biobank for VAT^ and summary results data for the skeletal outcomes. A genome-wide association (GWA) analysis was performed in the UK Biobank (n = 396,612) to identify instrumental variables of VAT^ (Additional File 3: Method S1). BMD-associated SNPs were identified from 3 separate GWAS summary statistics of European participants’ femoral neck bone mineral density (FN-BMD, n = 32,735), lumbar spine bone mineral density (LS-BMD, n = 28,498), and forearm bone mineral density (FA-BMD, n = 8143) [30]. For fractures, we used summary GWA results of hip fractures (the number of cases = 3,983; the number of controls = 211,460; GWAS ID: 'finn-b-ST19_FRACT_FEMUR') and vertebrae fractures (the number of cases = 2,859; the number of controls = 212,839; GWAS ID: ' finn-b-ST19_FRACT_LUMBAR_SPINE_PELVIS') from the FinnGen Biobank and the largest GWA results of total fractures from a meta-analysis of 25 cohorts [31] (the number of cases = 37,857; the number of controls = 227,116). The random-effect inverse-variance weighted (IVW) method was applied as the primary analysis [32]. We also performed MR Egger [33], weighted median [34], and mode-based regressions as sensitivity analyses. MR analyses were performed using the R ‘TwoSampleMR’ package [35].

## Results

### Baseline characteristics

The current study included 198,000 men (50.05%) and 197,612 women (49.95%) (Table 1). Compared to those with a lower VAT^, participants with a higher VAT^ were older and more likely to be current smokers, less likely to be drinkers, and had higher levels of BMI and lean mass, lower physical activity levels, and poorer baseline overall health. Furthermore, the prevalence of diabetes, CVD, and cancer was higher among higher VAT^ participants than among lower VAT^ participants. The distributions of the variables predicting VAT^ according to the gender-specific quartiles of VAT^ are presented in Additional file 1: Table S1. Furthermore, the baseline characteristics by sex are presented in Additional file 1: Table S2.

**Table 1:** Baseline characteristics of study participants by quartiles of VAT^ mass

	Men **(**n = 198,000**)**				Women (n = 197,612)			
	Q1 < 1133 g	Q2 1133–1651 g	Q3 1652–2252 g	Q4 > 2252 g	Q1 < 400 g	Q2 400–691 g	Q3 692–1079 g	Q4 > 1079 g
Age (year)	55.0 (8.4)	56.7 (8.2)	57.8 (7.8)	58.7 (7.4)	52.6 (8.1)	56.7 (7.8)	58.4 (7.5)	58.9 (7.3)
Post-menopause (%)	–	–	–	–	53.5	74.9	81.6	83.3
Household income (£)* (%)								
< 18,000	15.0	15.1	17.5	22.6	13.3	17.9	21.8	26.8
18,000–30,999	20.7	21.6	22.6	23.1	19.0	21.8	23.7	23.3
31,000–51,999	25.3	25.4	24.2	22.3	24.2	22.3	20.2	18.2
52,000–100,000	22.7	21.6	19.7	16.1	22.4	17.0	12.7	10.3
> 100,000	6.5	6.1	5.0	3.8	7.2	4.1	2.9	1.9
BMI (kg/m^2^)	23.5 (1.9)	26.3 (1.5)	28.5 (1.7)	33.1 (3.7)	22.2 (2.1)	24.8 (2.1)	27.5 (2.5)	33.1 (4.7)
VAT^ mass (g)	711.7 (364.3)	1387.2 (147.0)	1916.2 (169.9)	2913.4 (645.4)	214.4 (156.7)	542.6 (83.2)	886.9 (110.5)	1555.9 (460.2)
Lean mass (kg)	59.3 (6.3)	61.8 (6.2)	64.3 (6.3)	69.9 (7.6)	42.3 (3.8)	42.8 (3.8)	44.2 (3.9)	48.6 (5.3)
Smoking status (%)								
Never	57.6	51.8	46.0	39.7	64.5	60.7	57.6	53.8
Previous	28.4	36.2	42.4	48.8	26.6	30.8	33.7	37.0
Current	13.8	11.7	11.2	10.9	8.7	8.3	8.4	8.8
Alcohol consumption (%)								
Less than 3 times/month	20.3	17.5	18.8	24.5	28.6	30.2	33.8	45.0
1–2 times/week	25.8	25.9	25.9	27.6	27.1	26.8	27.1	25.5
More than 3 times/week	53.9	56.5	55.2	47.8	44.3	42.9	39.1	29.4
Physical activity (MET-min/week)	3211.9 (3111.2)	2943.7 (2949.2)	2760.8 (2913.2)	2404.0 (2774.5)	2821.3 (2569.8)	2630.3 (2468.0)	2492.6 (2426.3)	2158.3 (2318.7)
Calcium user (%)	1.0	0.7	0.7	0.7	3.9	3.6	3.2	2.8
Vitamin D user (%)	1.0	0.8	0.6	0.7	2.9	2.8	2.6	2.0
Baseline overall health rating (%)								
Excellent	26.2	18.8	12.5	6.5	28.3	21.6	15.5	8.2
Good	57.0	60.9	59.5	48.9	59.7	63.4	63.9	55.6
Fair	13.9	17.3	23.5	34.6	10.1	13.0	17.8	29.1
Poor	2.7	2.7	4.1	9.4	1.6	1.8	2.5	6.6
T2D (%)	2.1	3.4	5.8	14.3	1.0	1.3	2.1	8.3
CVD (%)	4.4	6.5	9.1	13.7	1.2	2.0	3.4	5.9
Cancer (%)	5.7	5.9	6.5	6.9	7.1	8.6	9.5	9.9

Values are mean (SD) or percentage (%). *£1.00 = $1.30, €1.20. VAT^, predicted values of VAT (visceral adipose tissue) mass

*BMI* body mass index; *MET* metabolic equivalent task; *T2D* type 2 diabetes; *CVD* cardiovascular disease; and *SD* standard deviation

### Observational analysis of VAT^ and BMD

**Fig. 2:**
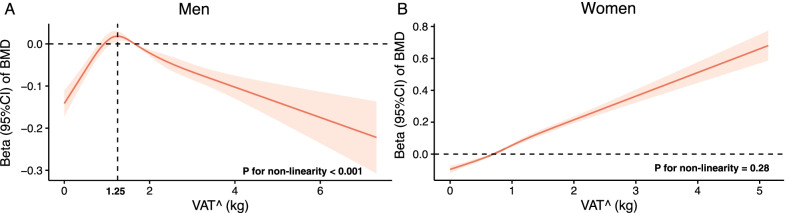
Association of VAT^ with heel BMD using restricted cubic splines. Betas are indicated by solid lines and 95% CIs by shaded areas. The reference point was the median of VAT^ in men (1.65 kg) and women (0.70 kg), separately, with knots placed at the 5th, 25th, 50th, 75th, and 95th centiles of each VAT^ distribution. All models were adjusted for age, household income, lean mass, standing height, smoking status, alcohol consumption, physical activity, calcium supplement use, vitamin D supplement use, overall health rating, diabetes, cardiovascular disease, cancer, and for women, menopausal status and use of hormone replacement therapy. *VAT*^ predicted values of VAT (visceral adipose tissue) mass; *BMD* bone mineral density; and *CI* confidence interval

From the restricted cubic splines regression analyses, in men, we observed an inverted U-shaped association between VAT^ and heel BMD (*P* for nonlinearity < 0.001, Fig. 2A), in which the turning point of VAT^ was around 1.25 kg (the corresponding BMI in this population was 25.7 kg/m^2^). Per kg increase in VAT^ was associated with 0.13 standard deviation (SD) increase in z-transformed heel BMD (*P* = 1.5 × 10^−16^) among men with lower amounts of VAT^ (< 1.25 kg); while associated with 0.05 SD decrease in heel BMD (*P* = 1.3 × 10^−15^) among men with higher amounts of VAT^ (≥ 1.25 kg). In women, per kg increase in VAT^ was associated with 0.16 SD increase in heel BMD with a significant linear trend (*P* for nonlinearity = 0.28, Fig. 2B). Consistently, we observed similar results when stratified by BMI (Table 2 and Additional file 1: Table S4). VAT^ was positively associated with heel BMD levels in men with BMI < 25 kg/m^2^ (β = 0.115, *P* = 1.2 × 10^−20^). However, in men with BMI ≥ 25 kg/m^2^, VAT^ was inversely associated with heel BMD (β = -0.045, *P* = 3.4 × 10^−18^; *P* for VAT^-BMI interaction = 5.9 × 10^−31^, Table 2). In women, VAT^ was monotonically associated with an increase in heel BMD regardless of their BMI levels (Table 2 and Additional file 1: Table S4). In the sensitivity analysis, after excluding participants with T2D, CVD, or cancer at baseline (n = 72,405), we got similar results (Additional file 1: Table S5). In a secondary analysis, we assessed the association between DXA-derived VAT and BMD in a subgroup of participants who underwent DXA examination (n = 5,109). The results (Additional file 1: Table S6) were highly consistent with the main results.

**Table 2:** Associations of VAT^ with heel BMD in men and women by BMI categories

Subgroup	Participants	Beta ± SE	*P*	*P* for VAT^-BMI interaction
*Men*				
All	190,836	− 0.008 ± 0.004	0.06	
BMI < 25 kg/m^2^	48,309	0.115 ± 0.012	1.2 × 10^−20^	5.9 × 10^−31^
BMI ≥ 25 kg/m^2^	142,527	− 0.045 ± 0.005	3.4 × 10^−18^	
*Women*				
All	193,592	0.161 ± 0.006	1.2 × 10^−136^	
BMI < 25 kg/m^2^	79,783	0.217 ± 0.017	7.8 × 10^−37^	0.06
BMI ≥ 25 kg/m^2^	113,809	0.125 ± 0.008	9.4 × 10^−56^	

Beta ± SE was estimated from the multivariable linear regression model. All models were adjusted for age, household income, lean mass, standing height, smoking status, alcohol consumption, physical activity, calcium supplement use, vitamin D supplement use, overall health rating, diabetes, cardiovascular disease, cancer, and for women, menopause status and use of hormone replacement therapy. *VAT*^ predicted values of VAT (visceral adipose tissue) mass; *BMD* bone mineral density; *BMI* body mass index; *SE* standard error. *P* for VAT^-sex interaction = 8.4 × 10^−51^

### Observational analysis of VAT^ and incident fractures

**Fig. 3:**
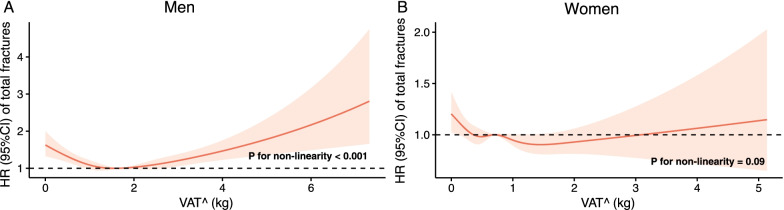
Association of VAT^ with total fracture risk using restricted cubic splines. HRs are indicated by solid lines and 95% CIs by shaded areas. The reference point was the median of VAT^ in men (1.67 kg) and women (0.69 kg), separately, with knots placed at the 5th, 25th, 50th, 75th, and 95th centiles of each VAT^ distribution. All models were adjusted for age, household income, lean mass, standing height, smoking status, alcohol consumption, physical activity, calcium supplement use, vitamin D supplement use, overall health rating, diabetes, cardiovascular disease, cancer, and for women, menopausal status and use of hormone replacement therapy. *VAT*^ predicted values of VAT (visceral adipose tissue) mass; *HR* hazard ratio; and *CI* confidence interval

During approximately 2,836,667 person-years of follow-up (median [IQR] follow-up, 8.2 [7.5–8.9] years), 9,688 incident total fractures, 1,290 incident hip fractures, 620 incident vertebrae fractures, and 8,199 incident other fractures were documented among 355,156 participants without a history of fracture at baseline (Table 3 and Additional file 1: Table S7). From the restricted cubic splines regression analyses, we observed a significant non-linear (U-shaped) association between VAT^ and total fracture risk in men only (*P* for nonlinearity < 0.001 in men, *P* for nonlinearity = 0.09 in women, Fig. 3A, B). Similarly, we observed significant non-linear relations of VAT^ with the risk of other fractures (*P* for nonlinearity < 0.001, Additional File 2: Fig. S4) in men only. Modeling as a continuous variable, VAT^ was only associated with the lower risk of hip fractures in the overall men population (*P* = 0.002, Table 3) and the overall women population (*P* = 0.02, Table 3). However, after excluding participants with T2D, CVD, or cancer at baseline(n = 66,815), the association was attenuated to non-significant (both *P* > 0.08, Additional file 1: Table S8). Similarly, in lean men with BMI < 25 kg/m^2^, higher VAT^ was associated with lower risks of total fractures (HR [95%CI], 0.80 [0.68–0.94]) and hip fractures (0.64 [0.50–0.81]), while in men with BMI ≥ 25 kg/m^2^, an adverse effect was observed (1.10 [1.02–1.18] for total fractures; 1.10 [1.01–1.19] for other fractures; both *P* for VAT^-BMI interaction < 0.05, Table 3). No significant association was found for any site of fractures in either women with BMI < 25 kg/m^2^ or women with BMI ≥ 25 kg/m^2^ (all *P* > 0.05, Table 3). To reduce potential collider bias, we also presented the results of the age-adjusted model and another minimally-adjusted model (Table S7). The inverse associations of VAT^ with the risk of hip fractures in men with BMI < 25 kg/m^2^ were consistent with that in the final model (Table S7 and Table 3).

**Table 3:** Associations of VAT^ with incident fractures in men and women by BMI categories

Outcome	Subgroup	Cases/participants	HR (95%CI)	*P*	*P* for VAT^-BMI interaction
*Men*					
Total fractures	All	3716/179,368	1.01 (0.95, 1.07)	0.75	
	BMI < 25 kg/m^2^	1028/44,469	0.82 (0.71, 0.95)	0.007	2.7 × 10^−4^
	BMI ≥ 25 kg/m^2^	2688/134,899	1.10 (1.02, 1.18)	0.02	
Hip fractures	All	512/179,368	0.78 (0.67, 0.91)	0.002	
	BMI < 25 kg/m^2^	188/44,469	0.64 (0.50, 0.81)	2.9 × 10^−4^	5.7 × 10^−3^
	BMI ≥ 25 kg/m^2^	324/134,899	0.94 (0.76, 1.16)	0.55	
Vertebrae fractures	All	335/179,368	1.17 (0.96, 1.43)	0.12	
	BMI < 25 kg/m^2^	74/44,469	1.07 (0.58, 1.99)	0.82	0.50
	BMI ≥ 25 kg/m^2^	261/134,899	1.19 (0.94,1.50)	0.15	
Other fractures	All	3074/179,368	1.03 (0.96, 1.10)	0.45	
	BMI < 25 kg/m^2^	833/44,469	0.86 (0.72, 1.01)	0.07	6.8 × 10^−3^
	BMI ≥ 25 kg/m^2^	2241/134,899	1.10 (1.01, 1.19)	0.02	
*Women*					
Total fractures	All	5972/175,788	0.93 (0.86, 1.01)	0.08	
	BMI < 25 kg/m^2^	2458/72,917	0.95 (0.78, 1.15)	0.60	0.91
	BMI ≥ 25 kg/m^2^	3514/102,871	0.99 (0.89, 1.10)	0.85	
Hip fractures	All	778/175,788	0.78 (0.63, 0.96)	0.02	
	BMI < 25 kg/m^2^	361/72,917	0.89 (0.58,1.36)	0.59	0.87
	BMI ≥ 25 kg/m^2^	417/102,871	0.91 (0.67, 1.22)	0.52	
Vertebrae fractures	All	285/175,788	0.95 (0.67, 1.36)	0.79	
	BMI < 25 kg/m^2^	122/72,917	0.88 (0.41, 1.89)	0.73	0.41
	BMI ≥ 25 kg/m^2^	163/102,871	1.21 (0.78,1.89)	0.40	
Other fractures	All	5125/175,788	0.97 (0.89, 1.05)	0.44	
	BMI < 25 kg/m^2^	2063/72,917	0.96 (0.78, 1.20)	0.75	0.92
	BMI ≥ 25 kg/m^2^	3062/102,871	1.00 (0.89, 1.11)	0.95	

HR (95% CI) was estimated from the Cox proportional hazards model. All models were adjusted for age, household income, lean mass, standing height, smoking status, alcohol consumption, physical activity, calcium supplement use, vitamin D supplement use, overall health rating, diabetes, cardiovascular disease, cancer, and for women, menopause status and use of hormone replacement therapy. *VAT*^, predicted values of VAT (visceral adipose tissue) mass; *BMI* body mass index; *HR* hazard ratio; and *CI* confidence interval. *P* for VAT^-sex interaction = 1.9 × 10^−4^ for total fractures; 0.06 for hip fractures; 0.39 for vertebrae fractures; 1.5 × 10^−4^ for other fractures

### Two-sample MR analysis

The MR estimates from different methods of assessing the causal effect of VAT^ on BMDs were presented in Table 4. The results demonstrated that genetically predicted VAT^ was not associated with the BMDs. With 1 kg increase of VAT^, the MR estimates from the IVW method were β − 0.045 (95%CI, − 0.127 to 0.037) for FN-BMD, − 0.062 (95%CI, − 0.157 to 0.033) for LS-BMD, and 0.023 (95%CI, − 0.116 to 0.163) for FA-BMD. In sensitivity analyses, consistent results were observed from weighted median, MR-Egger, and mode-based methods (Table 4). For fractures, across all MR methods, we did not observe a significant causal association between VAT^ and fractures either (Table 5). IVW odds ratio (OR) were 1.03 (95%CI, 0.93 to 1.15) for total fractures, 1.02 (95%CI, 0.84 to 1.24) for hip fractures, and 1.06 (95%CI, 0.85 to 1.32) for vertebrae fractures. Furthermore, MR analyses without excluding LM-associated SNPs suggested null causal relationships between VAT^ with either BMDs or fractures (Additional file 1: Table S11-12). In addition, no horizontal pleiotropy was detected for all MR analyses (Additional file 1: Table S13).

**Table 4:** MR estimates of the causal effects of VAT^ on BMDs

Method	FN-BMD		LS-BMD		FA-BMD	
	Beta ± SE	*P*	Beta ± SE	*P*	Beta ± SE	*P*
MR Egger	0.029 ± 0.170	0.86	− 0.105 ± 0.196	0.59	0.271 ± 0.286	0.34
Weighted median	− 0.030 ± 0.049	0.53	− 0.039 ± 0.058	0.50	0.117 ± 0.098	0.24
Inverse variance weighted	− 0.045 ± 0.042	0.29	− 0.062 ± 0.048	0.20	0.023 ± 0.071	0.75
Simple mode	0.108 ± 0.153	0.48	0.000 ± 0.186	1.00	0.213 ± 0.314	0.50
Weighted mode	− 0.067 ± 0.144	0.64	− 0.019 ± 0.181	0.92	0.213 ± 0.302	0.48

*MR* mendelian randomization; *VAT*^ predicted values of VAT (visceral adipose tissue) mass; *BMD* bone mineral density; *FN-BMD* femoral neck BMD; *LS-BMD* lumbar spine BMD; *FA-BMD* forearm BMD; *SE* standard error

**Table 5:** MR estimates of the causal effects of VAT^ on fractures

Method	Total fractures		Hip fractures		Vertebrae fractures	
	OR (95% CI)	*P*	OR (95% CI)	*P*	OR (95% CI)	*P*
MR Egger	0.67 (0.41, 1.10)	0.12	0.71 (0.32, 1.55)	0.39	0.93 (0.39, 2.26)	0.88
Weighted median	1.06 (0.93, 1.20)	0.42	0.99 (0.74, 1.32)	0.93	1.00 (0.73, 1.38)	0.98
Inverse variance weighted	1.03 (0.93, 1.15)	0.57	1.02 (0.84, 1.24)	0.83	1.06 (0.85, 1.32)	0.60
Simple mode	1.07 (0.68, 1.70)	0.77	0.87 (0.34, 2.24)	0.77	1.41 (0.48, 4.09)	0.53
Weighted mode	1.18 (0.77, 1.82)	0.44	0.92 (0.38, 2.23)	0.86	1.30 (0.48, 3.49)	0.61

*MR* mendelian randomization; *VAT*^ predicted values of VAT (visceral adipose tissue) mass; *OR* odds ratio; and *CI* confidence interval

## Discussion

Our study showed an inverted U-shaped association between VAT^ and heel BMD in men, while in women, the association was positively linear. In addition, we found significantly non-linear (U-shaped) relations between VAT^ with total fractures, hip fractures, and other fractures in men only. Subgroup analyses suggested that these associations were likely to be modulated by adiposity status especially in men. Two-sample MR analyses showed no causal association between VAT^ and skeletal outcomes.

Observational studies have reported controversial findings on the associations of VAT with skeletal outcomes [14, 36–39]. In line with our results in men with BMI ≥ 25 kg/m^2^, studies of obese men found that VAT was a negative predictor of bone microarchitecture and mechanical properties [38]. In contrast, the Framingham Osteoporosis Study found that higher amounts of VAT were associated with greater BMD and better microstructure of the peripheral skeleton, but the associations were not significant after adjustment for BMI or body weight [14], which was partially in support of our findings from women and men with BMI < 25 kg/m^2^. We also tried to adjust for BMI or bodyweight instead of lean mass and got consistent results. However, studies from Chinese adults reported that there was no correlation between VAT and BMD [39]. Though adjustment for potential confounding factors, the above cross-sectional analysis between VAT and BMD limited the causal inference. We also analyzed the association of VAT^ with incident fractures, and found that overall there were no significant associations among either men or women. Congruent with our results, a previous case-cohort study with a total of 252 fracture cases and 497 non-cases found there was no significant relationship between VAT and incident fractures [37]. However, previous studies did not explore the nonlinear relationship between VAT and skeletal outcomes [14, 36, 40, 41]. We found there were significant nonlinear associations of VAT^ with heel BMD and total fracture risk among men, with an inverse association at the lower range and a positive association at the higher range of VAT^. In summary, the relationship between VAT and skeletal outcomes was complex, and further studies in different populations are needed to validate our findings.

Furthermore, the interaction between VAT^ and adiposity status on skeletal outcomes was significant in this study, especially in men, which was similar to the results from the nonlinear association analyses. These sex-dependent associations can be attributed to the differences in the sex hormone levels in men and women [42]. Sex hormones play a major role in the growth and maintenance of the skeletal system [43], and have pronounced effects on adipose tissue [44]. Although the causal association between VAT and BMD remains unclear, previous studies suggested that adipose tissue could increase bone mass due to physical weight-bearing, while also imposing an adverse effect on BMD through the production of hormones and adipokines by adipocytes [45, 46]. From this perspective, it appears biologically plausible that the association between VAT and BMD may vary according to the amounts of VAT or by different adiposity statuses.

We were not aware of any previous MR study directly assessing the association between VAT and skeletal outcomes. In the present study, VAT^ showed no causal effect on skeletal outcomes. Consistent with our findings, an MR study found that BMI-adjusted waist circumference (a proxy for central fat distribution) was not correlated with BMD, suggesting that the effect of fat distribution might be neutral [47]. Similarly, Ma et al*.* recently conducted MR analyses and reported that BMI was causally associated with BMD but not associated with fracture. Moreover, they also found that the waist-to-hip ratio adjusted for BMI, hip circumference adjusted for BMI and waist circumference adjusted for BMI were not related to BMD or fracture occurrence [5]. In summary, these results did not support a causal relationship between VAT and skeletal outcomes, although further evidence is warranted to verify the reliability of our findings.

There were several strengths of our study. First, it was the largest population-based study to date to explore the association between VAT^ with BMD and fracture risk. The large sample size and the detailed information enabled us to conduct comprehensive statistical adjustments in the observational analyses. Second, we explored the potential nonlinear relationship of VAT^ with heel BMD and fracture risk. Besides, this was the first study to directly explore the potential causal association of VAT with DXA-derived BMD and fracture by MR analysis. Up to now, there only existed a few studies using surrogate-VAT (e.g., BMI-adjusted waist circumference [47]) to make causal inferences.

Several potential limitations should be acknowledged. Firstly, VAT was predicted rather than directly measured in the study, but previous studies have suggested that the prediction model for VAT had high coefficients of determination (R^2^ = 0.76) [17]. Secondly, the participants were White British from UK Biobank in our study. Therefore, the generalization of the study findings to other populations should be exercised with caution. Thirdly, for the MR analysis, we should acknowledge the slight sample overlap between the exposure and outcome GWAS datasets. However, we used powerful instruments to estimate the associations [48] between the exposures and the outcomes. Therefore, any sample overlap should not significantly bias our findings. Moreover, we used the largest GWAS datasets available for total fractures, which enabled adequate power to estimate the association between VAT^ and skeletal outcomes.

## Conclusions

Findings from this study demonstrated gender-dependent associations of VAT^ with BMD and fracture risk, with the association in men being modified by adiposity. Evidence of causality was not observed, suggesting that the observational association of VAT^ with BMD and fracture risk could be the result of confounding.

## Supplementary Information

**Additional file 1: Table 1**. Baseline characteristics of the final application datasets by quartiles of VAT^ mass. **Table 2**. Baseline characteristics of the final application datasets by sex. **Table 3**. ICD-10 codes for fracture definition in the UK biobank. **Table 4**. Associations of VAT^ with heel BMD in men and women by BMI categories. **Table 5**. Associations of VAT^ with heel BMD in men and women by BMI categories after excluding participants with T2D, CVD or cancer at baseline. **Table 6**. Association of DXA-derived VAT mass with DXA-derived BMD among participants by BMI categories. **Table 7**. Associations of VAT^ with incident fractures in men and women by BMI categories. **Table 8**. Associations of VAT^ with incident fractures in men and women by BMI categories after excluding participants with T2D, CVD or cancer at baseline. **Table 9**. Schoenfeld residuals test of the Cox models. **Table 10**. Genetic variants associated with VAT^ in the UK Biobank. **Table 11**. MR estimates of the causal effects of VAT^ on DXA-derived BMD without excluding LM-associated SNPs. **Table 12**. MR estimates of the causal effects of VAT^ on fractures without excluding LM-associated SNPs. **Table 13**. MR Egger test of directional horizontal pleiotropy.

**Additional file 2: Fig. 1**. The selection of participants. **Figure 2**. Association of VAT^ with hip fractures risk using restricted cubic splines. **Figure 3**. Association of VAT^ with vertebrae fractures risk using restricted cubic splines. **Figure 4**. Association of VAT^ with other fractures risk using restricted cubic splines.

**Additional file 3:** **Method 1.** The selection of instrumental variables in MR analyses.

## Data Availability

The UK Biobank data are available from the UK Biobank on application (www.ukbiobank.ac.uk/).

## Abbreviations

BMD: Bone mineral density
BMI: Body mass index
CVD: Cardiovascular disease
DXA: Dual-energy X-ray absorptiometry
FA-BMD: Forearm bone mineral density
FN-BMD: Femoral neck bone mineral density
GWA: Genome-wide association
IVW: Inverse-variance weighted
LM: Lean mass
LS-BMD: Lumbar spine bone mineral density
MR: Mendelian randomization
T2D: Type 2 diabetes
VAT: Visceral adipose tissue
VAT^: Predicted value of VAT mass
