# Supplementary material for: Associations of visceral adipose tissue with bone mineral density and fracture: observational and Mendelian randomization studies
Source: Nutr Metab (Lond). 2022 Jul 12;19:45. doi: 10.1186/s12986-022-00680-6 (PMC9277855; doi:10.1186/s12986-022-00680-6)
Supplement: Supplementary file 2 — Additional file 2: Fig. 1. The selection of participants. Figure 2. Association of VAT^ with hip fractures risk using restricted cubic splines. Figure 3. Association of VAT^ with vertebrae fractures risk using restricted cubic splines. Figure 4. Association of VAT^ with other fractures risk using restricted cubic splines. [file 12986_2022_680_MOESM2_ESM.docx]

**
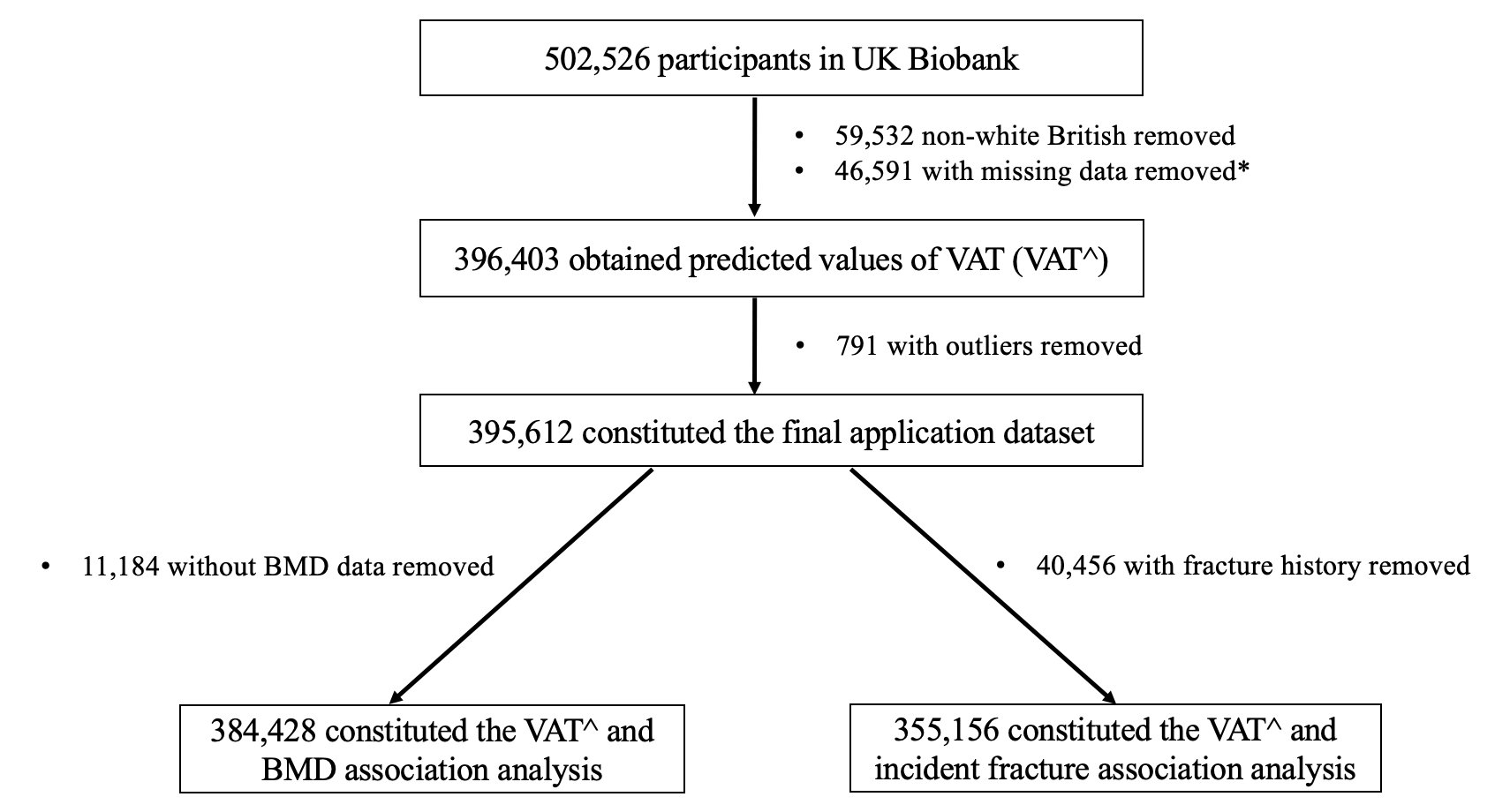
**

**Supplemental Fig. 1 The selection of participants.**

*****Participants had ‘Do not know’, ‘Prefer not to answer’ or ‘NA’ variables in the prediction model were set as missing the corresponding variables. VAT^, predicted values of VAT (visceral adipose tissue); BMD, bone mineral density.

**Supplemental Fig. 2 Association of VAT^ with hip fractures risk using restricted cubic splines.** The reference point was the median of VAT^ in men (1.67 kg) and women (0.69 kg), separately, with knots placed at 5th, 25th, 50th, 75th, and 95th centiles of each VAT^ distribution. All models were adjusted for age, household income, lean mass, standing height, smoking status, alcohol consumption, physical activity, calcium supplement use, vitamin D supplement use, overall health rating, diabetes, cardiovascular disease, cancer, and for women, menopausal status and use of hormone replacement therapy. VAT^, predicted values of VAT (visceral adipose tissue) mass; hazard ratio; and CI, confidence interval.

**Supplemental Fig. 3 Association of VAT^ with vertebrae fractures risk using restricted cubic splines.** The reference point was the median of VAT^ in men (1.67 kg) and women (0.69 kg), separately, with knots placed at 5th, 25th, 50th, 75th, and 95th centiles of each VAT^ distribution. All models were adjusted for age, household income, lean mass, standing height, smoking status, alcohol consumption, physical activity, calcium supplement use, vitamin D supplement use, overall health rating, diabetes, cardiovascular disease, cancer, and for women, menopausal status and use of hormone replacement therapy. VAT^, predicted values of VAT (visceral adipose tissue) mass; hazard ratio; and CI, confidence interval.

**Supplemental Fig. 4 Association of VAT^ with other fractures risk using restricted cubic splines.** The reference point was the median of VAT^ in men (1.67 kg) and women (0.69 kg), separately, with knots placed at 5th, 25th, 50th, 75th, and 95th centiles of each VAT^ distribution. All models were adjusted for age, household income, lean mass, standing height, smoking status, alcohol consumption, physical activity, calcium supplement use, vitamin D supplement use, overall health rating, diabetes, cardiovascular disease, cancer, and for women, menopausal status and use of hormone replacement therapy. VAT^, predicted values of VAT (visceral adipose tissue) mass; hazard ratio; and CI, confidence interval.
