# Supplementary material for: Associations of visceral adipose tissue with bone mineral density and fracture: observational and Mendelian randomization studies
Source: Nutr Metab (Lond). 2022 Jul 12;19:45. doi: 10.1186/s12986-022-00680-6 (PMC9277855; doi:10.1186/s12986-022-00680-6)
Supplement: Supplementary file 3 — Additional file 3: Method 1. The selection of instrumental variables in MR analyses. [file 12986_2022_680_MOESM3_ESM.docx]

**Supplemental Method 1. The selection of instrumental variables in MR analyses.**

We performed GWA analysis of VAT^ in the UK Biobank (n=395,612) (Fig. 1), in men and women separately, and then combined the results by using a fixed-effect meta-analysis. VAT^ was adjusted for age by the residual method and rank-transformed before the analysis^(1)^. The first 15 genetic principal components were included as covariates, together with a batch-effect variable to adjust for genotyping array (Axiom or BiLEVE)^(1)^. We identified 16,109 genome-wide significant SNPs (P < 5×10^−8^) for VAT^, of which 314 nearly independent lead SNPs were identified after clumping with a linkage disequilibrium threshold of R^2^=0.1 and a window of 2,000 kilobases^(1)^(Supplementary Table 10). Considering lean mass (LM) was an important confounder^(2)^, we excluded 100 LM-associated SNPs by the GWAS ID of ‘ukb-a-266’ in the MRC IEU OpenGWAS (https://gwas.mrcieu.ac.uk/datasets/), a database of genetic associations from GWAS summary datasets. In total, 214 SNPs were used as genetic instrumental variables (IVs) for VAT^.

For 214 VAT^-associated SNPs, 119 SNPs were not available in the summary statistics of total fractures. We replaced these unavailable SNPs with overlapping proxy SNPs in high linkage disequilibrium (r^2^ > 0.8) and found 86 proxy SNPs for total fractures. When harmonizing exposure and outcome data, 31 SNPs were further excluded for total fractures. Finally, 140 VAT^-associated SNPs were included as IVs for the analysis of total fractures (Fig. 1). Similarly, after replaced the unavailable SNPs with proxy SNPs and harmonized data, 210, 210, 207, 211 and 211 VAT^-associated SNPs were included as IVs for the analysis of FN-BMD, LS-BMD, FA-BMD, hip fractures and vertebrae fractures, respectively (Fig. 1).

All included SNPs have F statistics > 10, suggesting little instrument bias^(3)^

**References**

1. Karlsson T, Rask-Andersen M, Pan G, Höglund J, Wadelius C, Ek WE, et al. Contribution of genetics to visceral adiposity and its relation to cardiovascular and metabolic disease. Nat Med. 2019;25(9):1390-5.

2. Viardot A, Purtell L, Nguyen TV, Campbell LV. Relative Contributions of Lean and Fat Mass to Bone Mineral Density: Insight From Prader-Willi Syndrome. Front Endocrinol (Lausanne). 2018;9:480.

3. Hemani G, Zheng J, Elsworth B, Wade KH, Haberland V, Baird D, et al. The MR-Base platform supports systematic causal inference across the human phenome. Elife. 2018;7.
